# Manual and mechanical restraint and the hierarchy of coercive measures: Evidence or tradition?

**DOI:** 10.1192/j.eurpsy.2021.997

**Published:** 2021-08-13

**Authors:** M. Lynge, S. Dixen, K. Johansen, S. Düring, A. Parnas, J. Nordgaard

**Affiliations:** 1 Mental Health Center Amager, Mental Health Services in the Capital Region of Denmark, Copenhagen S, Denmark; 2 Competence Centre For Dual Diagnosis, Mental Health Services in the Capital Region of Denmark, Roskilde, Denmark; 3 Mental Health Center Glostrup, Mental Health Services in the Capital Region of Denmark, Copenhagen V, Denmark; 4 Mental Health Center Amager, Mental Health Services in the Capital Region of Denmark, Copenhagen V, Denmark

**Keywords:** Mechanical restraint, Patient experiences, coercion, Manual restraint

## Abstract

**Introduction:**

In the continuous work to reduce the use of coercion in the psychiatric care, attention in Denmark has especially been directed towards mechanical restraint, i.e. the use of belts to fixate patients to a bed. While the use of mechanical restraint is currently decreasing, increases in other types of coercive acts are observed (e.g., forced medication and hourly episodes of manual restraint). The use of manual restraint refers to mental health workers immobilizing a patient to avoid harm to self or others. Manual restraint is generally considered less intrusive to a patient’s autonomy than the use of mechanical restraint. However, no study has yet explored if it is actually experienced as such by the patients.

**Objectives:**

This study explores patients’ perspectives on manual and mechanical restraint, respectively.

**Methods:**

We are currently performing a qualitative interview study of 10 patients, who have been exposed to both types of coercion. The interviews will be transcribed verbatim and analysed for thematic content.

**Results:**

We expect to discover more nuanced perspectives of the intrusiveness of the different forms of coercion—perspectives that may challenge the assumption that one type of coercion is by default better than another. The study’s results will be presented.

**Conclusions:**

In this study, we only look at two types of coercion. More investigation into the differentiation of patients and ideal type of coercive measure is paramount to the ambitions of a better and more humanistic psychiatric care.

